# Fluorescein-derived carbon dots with chitin-targeting for ultrafast and superstable fluorescent imaging of fungi

**DOI:** 10.1515/nanoph-2022-0468

**Published:** 2022-09-29

**Authors:** Ao Liu, Yiqiao Chen, Biwen Yang, Zhouyi Guo, Luoqi Mo, Haolin Chen, Chenglong Tao, Chengkang Su, Zhiming Liu

**Affiliations:** MOE Key Laboratory of Laser Life Science & Guangdong Provincial Key Laboratory of Laser Life Science, College of Biophotonics, South China Normal University, Guangzhou 510631, China; Department of Hematology, The Seventh Affiliated Hospital, Sun Yat-sen University, Shenzhen 518107, China; Guangzhou Haokang Biotechnology Co., Ltd., Guangzhou 510660, China; Guangzhou Key Laboratory of Spectral Analysis and Functional Probes, College of Biophotonics, South China Normal University, Guangzhou 510631, China

**Keywords:** carbon dots, chitin-targeting, fluorescent brightener, fungi imaging, photoluminescence features

## Abstract

Fluorescence microscopy based on fluorochrome has been rapidly developed as the candidate for morphological identification of pathogenic fungi over recent years, offering superior rapidity and efficacy over traditional culture methods. However, the intrinsic quenching properties of fluorescein limit the clinical application of fluorescence imaging. Herein, we report a nano-strategy by converting a commercial fluorescein dye, fluorescent brightener-33 (FB-33), into carbon dots (FB-CDs) through a one-pot hydrothermal method. FB-CDs exhibit a chitin-targeting capacity allowing the selective recognition and ultrafast imaging of fungi within 30 s. The fluorescence quantum yield of FB-CDs is 51.6% which is 8.6-fold higher than that of commercial dye, FB-33. Moreover, FB-CDs also display superstable fluorescence signals under continuous intense light irradiation for 2 h and long-term storage for more than 2 months. The significantly improved photobleaching resistance meets the prolonged fluorescence observation and quantitative analysis of microbial samples. This work offers a novel nanoconversion strategy of commercial dyes for point-of-care testing of pathogenic organisms.

## 1 Introduction

The pandemic has refocused our attention on the risk of microbial infection [1, 2]. This concern is also accompanied by the abuse of broad-spectrum antibiotics, anti-tumor drugs, and immunosuppressants. Compared with others, fungal infection is more complicated due to the diverse fungal structures, as well as the imprecise clinical manifestations that may easily lead to missed diagnosis and misdiagnosis [3]. Thus, early detection and accurate identification of fungal pathogens is of great significance for clinical medication treatment and epidemiological research. To date, the traditional strategies for pathogen detection based on morphology and molecular biology involve morphological observation after long-term enrichment culture, polymerase chain reaction and serological tests, which are often limited by the expensive equipment, complex training requirements, long detection period, and false negatives [4–6]. Microscopic examination is the golden standard means for the clinical fungal diagnosis that requires only simple microscopic equipment, largely meeting the point-of-care testing (POCT) device needs [7, 8]. However, bright-field microscope is often difficult to identify pathogen in clinical sample with various impurities due to its low contrast.

Fluorescent labeling technique provides an effective way for microscopy imaging to enhance the contrast, achieving precise pathogen identification. Numerous commercial fluorescent molecules have been developed for fluorescent staining imaging to identify pathogenic microorganisms, such as fluorescent brighteners (FBs), fluorescein isothiocyanate (FITC), propidium iodide and rhodamine B [9–11]. Among that, FBs are typically diaminostilbene derivative compounds that are capable of specifically binding to b-linked fibrillar polymers on fungal cell walls via hydrogen bonds, such as cellulose and chitin [12, 13]. However, organic fluorescent dyes always suffer from intrinsic shortcomings such as low photostability, environmental instability, small photoluminescence quantum yield (PLQY), and poor biocompatibility [14], which are difficult for long-term storage and continuous microbial imaging. Nanotechnology brings new opportunities to overcome the above shortcomings [15–21]. Carbon dots (CDs), new type of fluorescent nanomaterials, have attracted widely attention owing to their tunable photoluminescence properties, good photostability, low cost, and excellent biocompatibility [22–26]. The optical features and biochemical activities of CDs can be easily shaped through facile synthetic strategies by using diverse precursors or doping with various elements [27–32]. Dye molecules are also adopted as the precursors to fabricate high-performance fluorescent CDs with high PLQYs [33–35]. For fungal imaging, CDs have been successfully utilized as fluorescent nanoprobes to distinguish the cellular survival and species in yeast and aspergillus [36–38]. However, the current CDs lack targeting capability so that a relative long sample preparation time (including staining and purification steps) is always needed, which is not conducive to the POCT of pathogens.

**Scheme 1:**
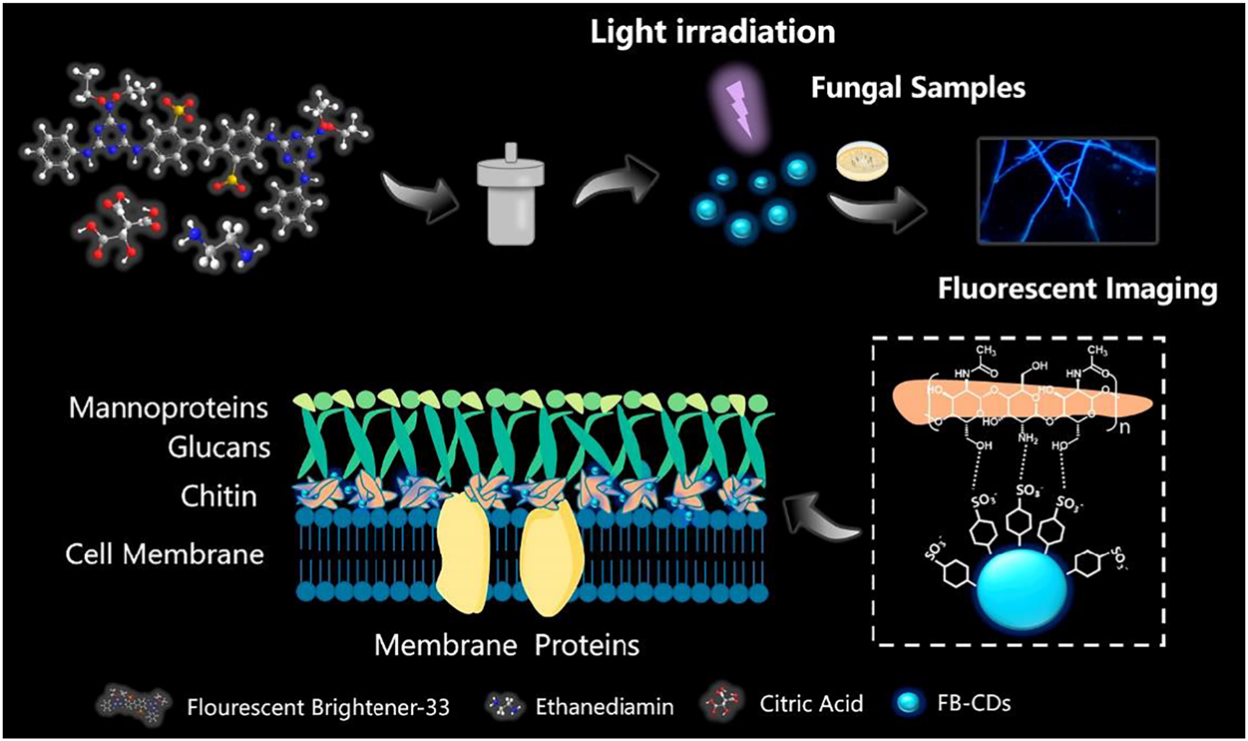
Schematic illustration of the synthesis of FB-CDs and their application for chitin-targeting fungal imaging.

Inspired by the structural plasticity of CDs, herein, we propose a nanostrategy for conversion of commercial FB-dyes in order to achieving fast, stable, and targeted fungal imaging. FB-derived CDs (FB-CDs) are prepared via one-step hydrothermal treatment using fluorescent brighteners-33 (FB-33), citric acid, and ethanediamine as the precursors (Scheme 1). The obtained FB-CDs exhibit a unique excitation-independent optical property with high PLQY of 51.6%, while the quantum yield of FB-33 is only 6.0%. The fluorescence signals of FB-CDs are also superstable under different application environments (such as long exposure time, long-term storage, and pH). The FB-derived CDs largely inherit the targeting capacity of FB-33 that can rapidly bind to chitin in fungal cell wall, which allows an ultrafast fluorescent imaging of pathogens without washing steps. Further combined with a cheap commercial microscope, we realize the efficient fluorescent identification of *Schizophyllum commune* and *Saccharomyces cerevisiae* within 30 s, demonstrating the great potential for POCT of pathogenic microbial infection.

## 2 Results and discussion

### 2.1 Characterization of FB-CDs

**Figure 1:**
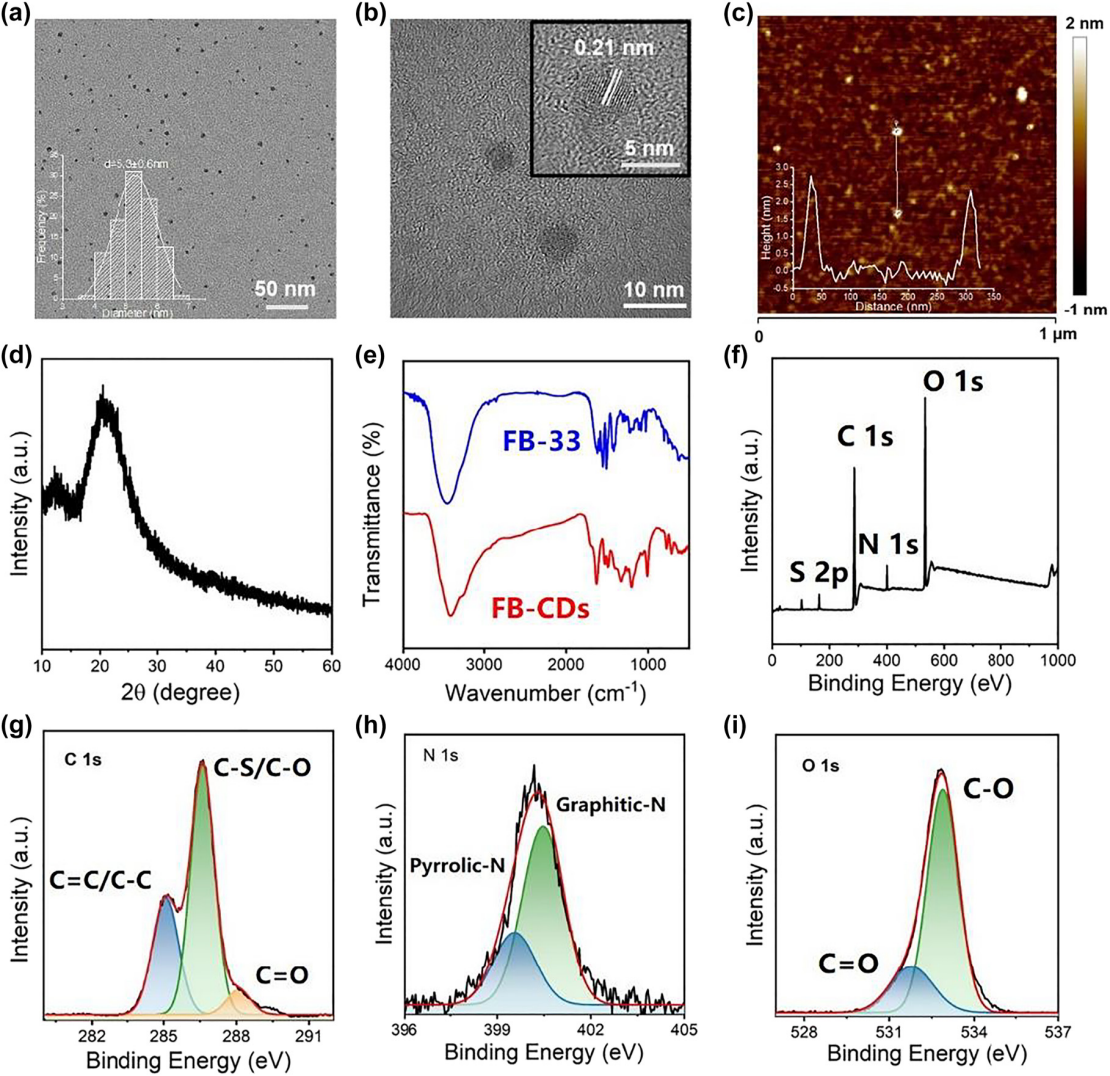
Characterization of FB-CDs. (a) and (b) TEM images of FB-CDs at different magnifications (Insets: (a) Particle size distribution and (b) the high-resolution TEM image of FB-CDs). (c) AFM image of FB-CDs, the inset showed the height profile along the line. (d) XRD pattern of FB-CDs. (e) FT-IR spectra of FB-CDs and FB-33, respectively. XPS analysis of FB-CDs: (f) survey scan, (g) C 1s, (h) N 1s, and (i) O 1s spectra.

The morphology and size distribution of FB-CDs were evaluated by transmission electron microscopy (TEM), which revealed that FB-CDs were uniformly dispersed particles with an average diameter of 5.3 nm (Figure 1a). The high-resolution TEM result showed that lattice spacing of 0.21 nm, which corresponded to typical <100> facet in graphite (Figure 1b). The atomic force microscopy (AFM) image reveals the height of CDs ranged from 2.5 to 3.0 nm (Figure 1c), indicating that CDs primarily consisted of a few layers of graphene-like plates [39, 40]. The X-ray diffraction (XRD) pattern displayed a broad peak centered at 23° (Figure 1d) corresponded to the typical peak of CDs as previously reported, which confirmed their high crystalline graphene structure [41]. The elemental composition and functional groups of FB-CDs were investigated by Fourier transform infrared (FT-IR) and X-ray photoelectron spectroscopy (XPS). As shown in Figure 1e, the absorption peaks centered at 1620, 1550, 3447 and 3408 cm^−1^ in the FT-IR spectrum of FB-CDs could be attributed to the stretching vibrations of C=O, C=C, O–H and N–H, respectively. The spectral signals at 1485 and 1195 cm^−1^ might come from the C–N and C–O stretching vibration of the thiotriazinone group in the CDs [42]. Similar spectral features at 1000, 771 and 600 cm^−1^, attributed to S=O, S–O and S–C stretching vibrations, respectively, was observed from the spectra of both FB-33 and FB-CDs, corresponding to the benzenesulfonic acid group on FB-33 molecule [35, 42, 43]. The full XPS spectrum implied that the FB-CDs included C, N, O, and S elements (Figure 1f). The curve of C 1s (Figure 1g) could be deconvoluted into three peaks including C–C/C=C (284.97 eV), C–S/C–O (286.60 eV), and C=O (288.10 eV), respectively [42]. The N 1s spectrum (Figure 1h) could be fitted into two peaks, which could be assigned to pyrrolic N (399.55 eV) and graphitic N (400.46 eV), respectively. The two peaks centered at 531.78 and 532.89 eV in the O 1s curve (Figure 1i) indicated the existence of C=O and C–O, respectively [39]. Therefore, the nanostructure of FB-CDs is probably consisted of a graphitized carbon core and the fluorescein-like surface functional groups.

### 2.2 Photoluminescence features of FB-CDs

**Figure 2:**
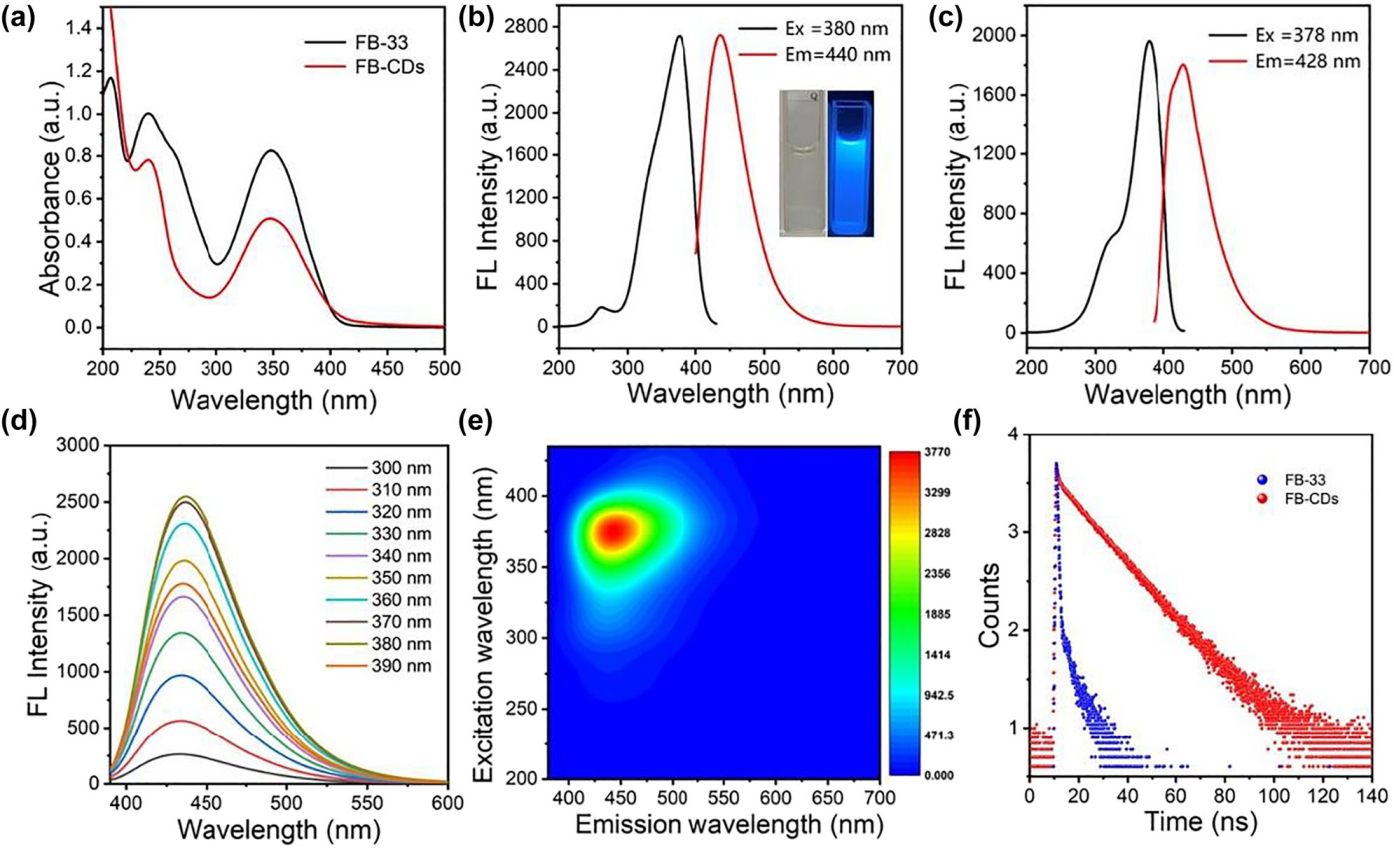
Optical properties of FB-CDs. (a) Ultraviolet–visible (UV–vis) spectrums of the FB-CDs and FB-33 aqueous solution (100 μg/mL). (b) Fluorescence maximum excitation and emission spectra of FB-CDs (100 μg/mL). Insets: Photographs of FB-CDs solution under daylight (left) and UV light irradiation (right). (c) Fluorescence maximum excitation and emission spectra of the FB-33 (100 μg/mL). (d) Fluorescence emission spectra of the FB-CDs collected at various excitation wavelengths. (e) Excitation-emission contour plot of FB-CDs. (f) Time-resolved fluorescence spectra of FB-33 and FB-CDs.

The Ultraviolet-visible (UV–vis) spectrum of the FB-CDs aqueous dispersion exhibited absorption peaks at 240 and 350 nm (Figure 2a) which were associated with the *π* = *π* electronic transition of C=C and the *n* − π* transition of surface groups (C=O and C=N, etc.), respectively [44]. While the absorption bands of FB-33 stood at 348 and 265 nm, which belonged to trans isomers and cis isomers of stilbene structure in FB-33, respectively. Then the photoluminescence property of the FB-CDs was investigated. As shown in Figure 2b, FB-CDs displayed fluorescence (FL) emission with the maximum excitation and emission peaks at 380 and 440 nm, respectively. The emission wavelength of FB-CDs was approximately red-shifted of 10 nm in comparison with that of FB-33 (Figure 2c), which may be attributed to the increased *sp* ^2^ domain size in CDs, leading to the decreased band gap [45]. The Stokes shift of FB-CDs was speculated to eliminate the FL self-quenching caused by self-absorption and significantly improve the FL photostability [46]. We further controlled the synthetic process to gain the optimal FL property of FB-CDs, and the hydrothermal temperature and time were finally determined to be 180 °C and 8 h, respectively (Figure S1). The FL signals of FB-CDs were obviously brighter than that of FB-33 under UV light irradiation at the same concentration (0.1 mg/mL), perhaps due to intersystem crossover transitions and cis-trans isomerization of FB-33 being restricted by the conjugation structure of carbon dots (Figure S2). FB-CDs also displayed an excitation-independent emission (Figure 2d), showing a good monochromaticity, which could also be confirmed by the excitation-emission contour plot (Figure 2e) and the three-dimensional fluorescence map (Figure S3).

The time-resolved fluorescence spectrum was examined as depicted in Figure 2f. The fluorescence lifetime of the FB-CDs was calculated to be 14.962 ns, which was much longer than 1.308 ns for FB-33, indicating the remarkably enhanced FL performance of FB-CDs. Using an absolute quantum yield measurement system, we also obtained the absolute PLQY value of FB-CDs to be 51.6%, while the PLQY values of FB-33 and dye-free CA-CDs were 6.0 and 13.7%, respectively (Figure S4). The PLQY of FB-CDs is also superior to other reported nanoagents (Table S1). FB-33 is triazine-stilbene derivative compound with low steric hindrance, which can make the molecule isomerize by rotating around the double bond easily in solution [47]. The trans-isomer (centered at 350 nm) of FB-33 is easily converted to nonfluorescent cis-isomer structure (270 nm) under UV irradiation (Figure S5), the pattern of UV–vis spectrum of FB-CDs showed no obvious change under the same UV irradiation. We speculate the nanoscale carbon-dot structure of FB-CDs may restrict the intersystem crossing, nonradiation transition and the cis-trans isomerization of fluorescein groups while vastly improving planar rigidity, fluorescence efficiency and lifetime, reducing the possibility of the excited state (S1) transitioning to the triplet state (T1) and the photosensitization reaction [48].

### 2.3 Photostability of FB-CDs

**Figure 3:**
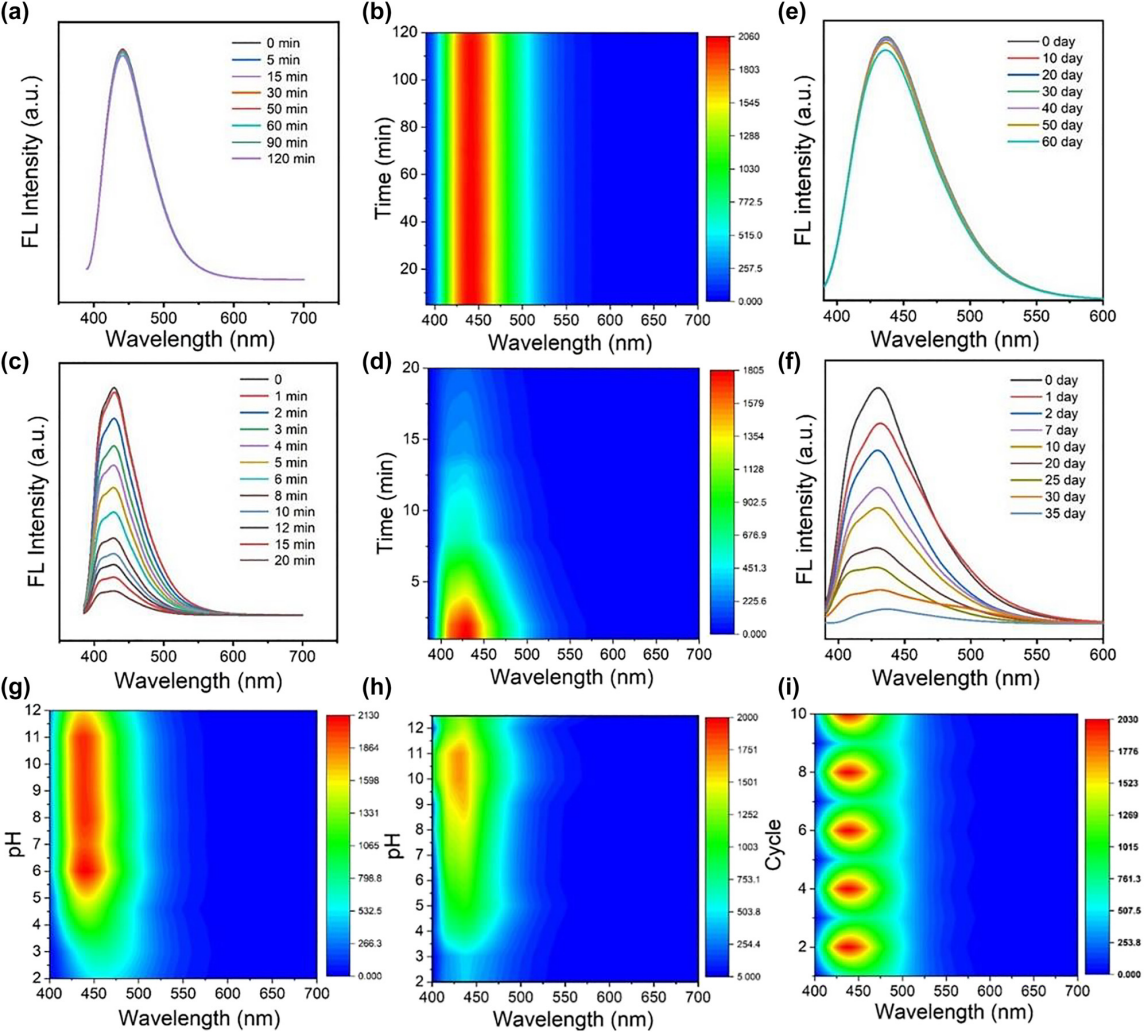
Fluorescence emission spectra and corresponding contour plots of FB-CDs (a, b) and FB-33 (c, d) as a function of UV irradiation time, respectively. Fluorescence spectra of (e) FB-CDs and (f) FB-33 after different storage times. Normalized color plots of fluorescence spectra of (g) FB-CDs and (h) FB-33 solutions at different pH values (from 2 to 12). (i) Normalized color plots of the reversible PL intensity of FB-CDs with varying pH between 3 and 11.

The optical stability of fluorescent probes is of great importance for their clinical applications. UV illumination time, storage time, and pH were selected as influencing factors to evaluate the photoluminescence stability of the FB-CDs. Figure 3a demonstrated the time-evolved fluorescence emission spectra of FB-CDs under intense UV illumination. It could be noticed that all the spectral lines were almost overlapped, revealing an superstable FL feature of FB-CDs during a continuous UV irradiation (Ex = 380 nm) for 2 h. The superstable FL signals could also be clearly visualized in the FL contour plot (Figure 3b). In addition, the photostability of FB-CDs dispersed in fetal bovine serum (FBS) and lysogeny broth (LB) medium were also examined, where no obvious quenching was observed after continuously UV exposure for 40 min (Figure S6). In marked contrast, the FL signals of FB-33 dramatically decreased along with the extension of UV irradiation (Ex = 378 nm) time, and was almost quenched after 20 min of UV exposure (Figure 3c and d). For storage stability, Figure 3e showed the strong FL signals of FB-CDs with almost constant intensity which decreased slightly after 60-day storage. While the fluorescence signals of FB-33 reduced significantly and were difficult to detect at day 35 (Figure 3f); we finally studied the fluorescence feature of the FB-CDs under various pH conditions. It could be observed that the pH stability of FB-CDs was obviously superior to that of FB-33, especially in the physiological pH range (Figures 3g and h and S7), indicating a better applicability of FB-CDs for biosensing. Furthermore, the pH-responsive characteristics of FB-CDs was reversible, where the FL signals of FB-CDs only showed minor decreased over ten consecutive cycles (Figure 3i). The data above imply the promising potential of FB-CDs as superstable FL nanoprobe against long-term light exposure and storage, as well as unfriendly detection environment.

### 2.4 Chitin-targeting capability of FB-CDs

**Figure 4:**
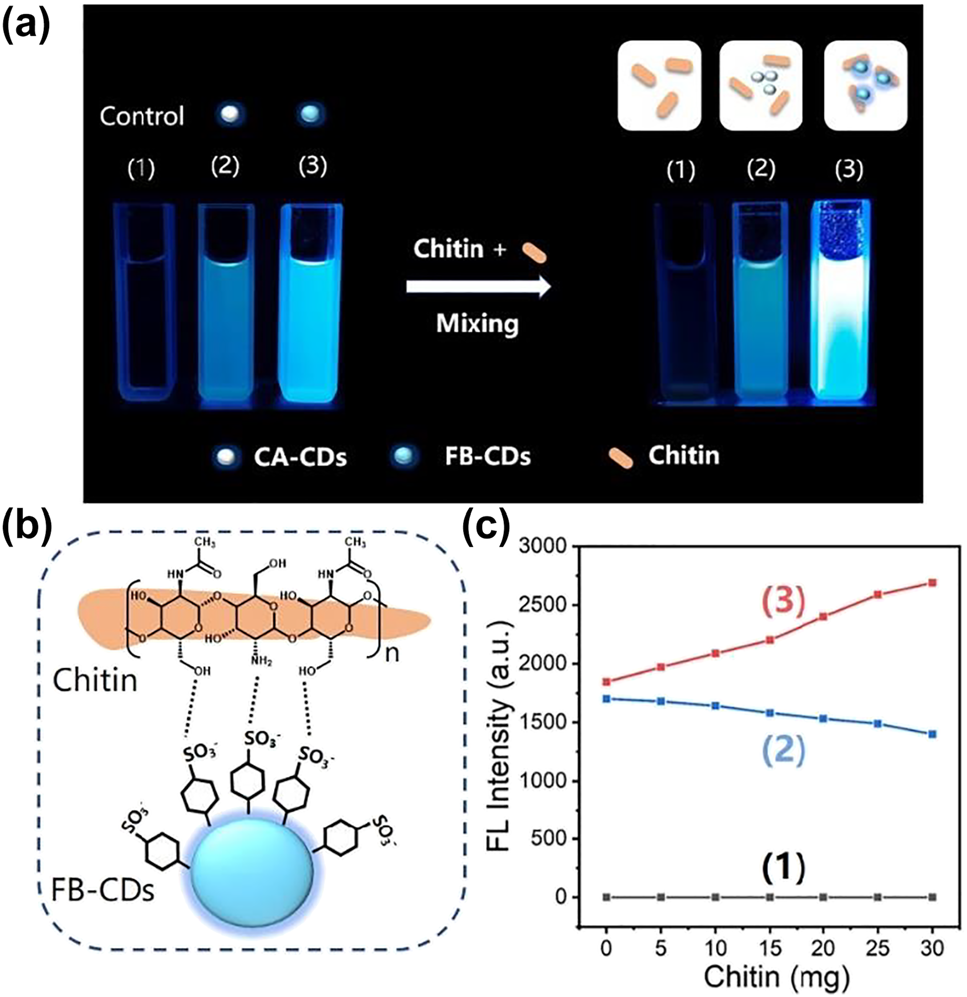
The chitin-targeting capability of FB-CDs. (a) FL images of (1) water (control), (2) CA-CDs, and (3) FB-CDs under UV irradiation. The CD solutions were compared at the same concentration. (b) Schematic illustration of the binding mechanism chitin to FB-CDs. (c) Fluorescence intensities of CD solutions after mixed with different chitin mass.

Chitin is a polysaccharide composed of repeated β-(1–4)-linked N-acetyl-D-glucosamine, which contains abundant hydrogen bond binding sites. The chitin-specific recognition ability of FBs has been confirmed by literature and widely clinical applications [49]. To investigate the chitin-targeting capability of FB-CDs, the *in tube* experiments were carried out according to previous work. The FL intensity of FB-33 showed a linearly positive correlation with its chitin-binding content (Figure S8), which confirmed the validity of this method [50, 51]. The changes of FL signals after grafting of chitin to FB-CDs or CA-CDs (FB-33 free) were displayed in Figure 4. It could be observed from the FL images that the brightness of CA-CDs solution hardly altered after adding of chitin, while the FL signals of FB-CDs solution after mixing with chitin became significantly stronger (Figure 4a). We deduced that a hydrogen bond interaction formed between chitin and the benzenesulfonic acid group on the surface of carbon core, leading to increasing FL intensity of FB-CDs (Figure 4b). Furthermore, the CDs solutions were mixed with different concentrations of chitin and the changes of their FL intensities were plotted in Figure 4c. The FL signals of CA-CDs gradually decreased with the increase of chitin mass, which might be ascribed to the aggregation-triggered FL quenching caused by the steric hindrance by increasing chitin. In contrast, the FL intensity of FB-CDs was proportional to the chitin mass, which was consistent with the phenomenon in FB-33 (Figure S8), indicating the chitin-targeting ability of FB-CDs. In addition, these chitin-targeted FL signals of FB-CDs could not be affected by cations (Figure S9).

**Figure 5:**
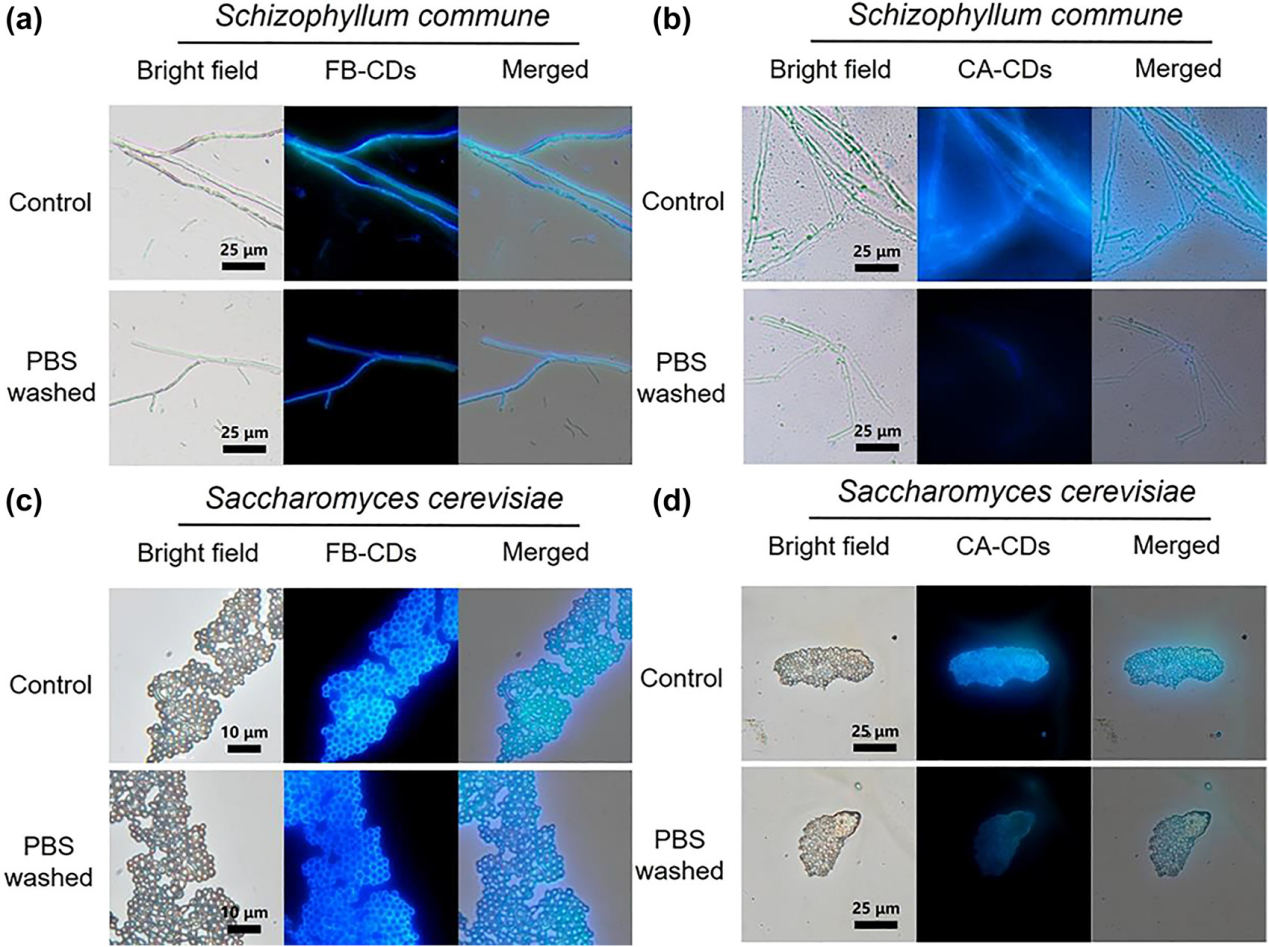
Chitin-targeted fungal fluorescence imaging. FL images of *S. commune* (a, b) and *S. cerevisiae* (c, d) using FB-CDs (a, c) and CA-CDs (b, d) as the nanoprobes, respectively.

The chitin-targeting capability of FB-CDs towards fungus was then studied using two typical fungal species, *S. commune* and *S. cerevisiae* (*S. cerevisiae*) as models. Figure 5 illustrated the FL images of fungi after incubation with CDs for 30 s with or without PBS washing. The FL image of *S. commune* stained with FB-CDs displayed a clear fungal contour with negligible background interference; and the intense FL signals could not be cleaned by two rounds of PBS (Figure 5a), indicating that FB-CDs were firmly anchored to the fungal cell wall. However, the FL image of *S. commune* after treated with CA-CDs under the same experimental condition showed a vague fungal outline (Figure 5b). After two times of PBS cleaning, the FL signals reduced significantly, and the fungi were almost indistinguishable, demonstrating that CA-CDs had been eluted. The disparate phenomena between FB-CDs and CA-CDs can be explained as the affinity of FB-CDs to chitin achieving the fungal imaging in short time. The comparative imaging experiment of FB-CDs and CA-CDs was further performed on *S. cerevisiae*, where similar results were acquired (Figure 5c and d), indicating the universality of chitin-targeting FB-CDs for multiple fungal species. In addition, human hepatoma (HepG2) cells, kind of mammalian cells without cell wall, were also adopted as the negative control to verify the imaging ability of FB-CDs. We could observe from Figure S10 that the FL image of HepG2 cells was obscure and poorly contrasted, although the cells were incubated with carbon dots for 10 min (much longer than 30 s for fungal imaging). Therefore, the fungus-specific, chitin-targeting feature of FB-CDs highlighted the potential of fluorescein-derived CDs for fast fungal imaging.

### 2.5 Ultrafast and superstable fungal imaging based on FB-CDs

For biomedical applications, the biocompatibility of fluorescence probe is of great importance. The cytotoxicity evaluation was carried out by incubating *S. cerevisiae* with different concentrations of FB-CDs, which revealed excellent cytocompatibility even the concentration of FB-CDs was up to 1 mg/mL (Figure S11). We also noticed that the cytotoxicity of FB-CDs was much lower than that of FB-33, suggesting a better feasibility for bioapplications. For fast bioimaging, the experimental parameters of FB-CDs were first optimized. The time- and dose-dependent FL images of *S. commune* were observed (Figures S12 and S13). It could be observed that the fungi treated with 100 μg/mL of FB-CDs for 30 s (wash-free) was enough to gain a credible result.

**Figure 6:**
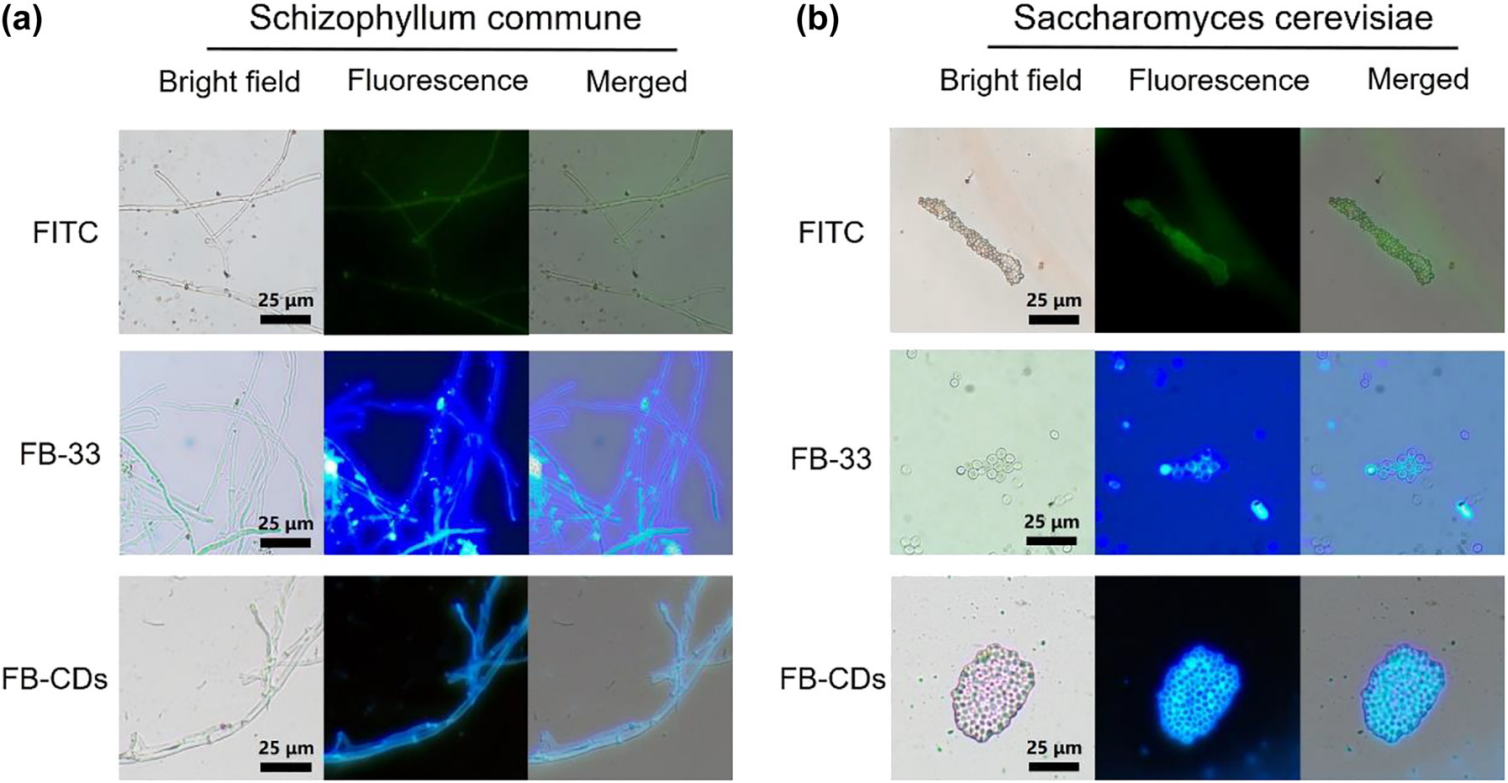
Ultrafast fluorescence imaging based on FB-CDs. Fluorescence images of (a) *S. commune* and (b) *S*. *cerevisiae* treated with FITC, FB-33 and FB-CDs at the same concentration (100 μg/mL) for 30 s.

The comparative fungal imaging experiments with FB-CDs and commercial dyes were further implemented. As shown in Figure 6a, the *S. commune* cells emitted strong fluorescence signals, and the septum of mycelium could be clearly distinguished after incubation with FB-CDs for 30 s. FL images with clear boundaries could also be observed in *S. cerevisiae* stained with FB-CDs (Figure 6b). Whereas, the morphologies of FB-33 labeled fungal cells were fuzzy with strong fluorescence background interference, which was consistent with the data reported in other studies [52, 53]. The fungal imaging results of FITC were similar to that of FB-33. The strong background in dye-based FL images is probably originated from the FL signals of discrete dye molecules under wash-free operation. While free FB-CDs without binding to fungi may result in aggregation and subsequently FL self-quenching, obtaining a low interference FL image. Therefore, the FL technique based on FB-CDs is capable of achieving reliable fungal imaging during an ultrafast process (within 30 s).

**Figure 7:**
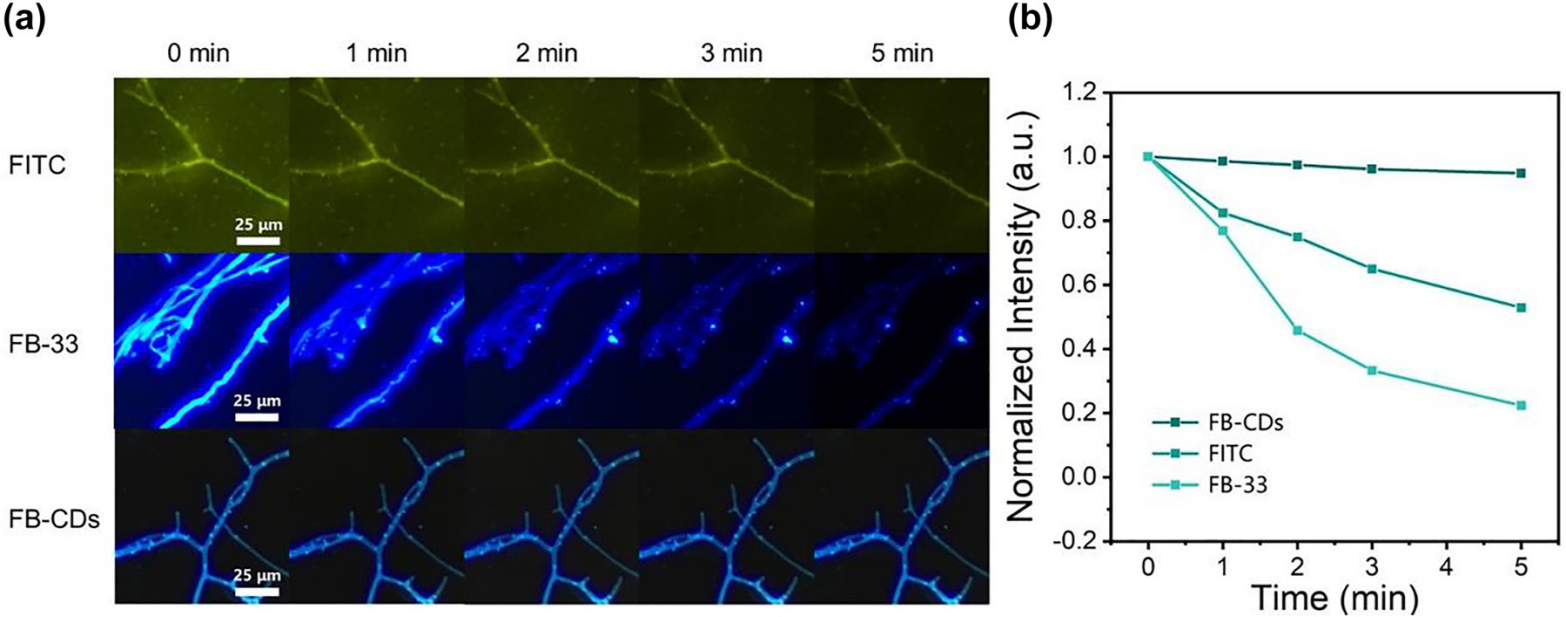
Photostability evaluation results of FB-CDs. (a) Fluorescence images of *S. commune* cells after being treated with FITC, FB-33, or FB-CDs captured every 1 min. (b) Corresponding fluorescence quenching curves calculated from the fungal images.

To further investigate the fluorescence stability of FB-CDs in fungus cells, time-lapse FL imaging of *S. commune* was carried out. Figure 7a illustrated the FL images of *S. commune* stained with fluorescence probes (FITC, FB-33 and FB-CDs) at the concentration of 100 μg/mL. During a 5 min of observation, the FL signals of FITC or FB-33 quenched dramatically. However, the FL signals in *S. commune* stained with FB-CDs remained nearly unchanged (Figure 7b); also, negligible background interference was noticed. And the FL signals in fungal image hardly attenuated after a continuous observation for 30 min (Figure S14), indicating a superstable fluorescence feature of FB-CDs for bioimaging. Table S2 also listed common fluorescent molecules for fungi imaging, revealing the better potential of FB-CDs for fungal imaging. FB-CDs-based FL imaging was finally carried out on the simulated clinical sample by mixing *S. commune* with human scurf. As shown in Figure S15, the fungal strains could be clearly observed under the fluorescent microscope, and the scurf itself did not produce significant background interference, demonstrating the potential of FB-CDS application in real pathological samples. The wash-free, fast, and accurate identification of fungal cells demonstrates that FB-CDs can be employed as a novel reliable fluorescent probe for clinical mycosis detection without considering fluorescence quenching.

## 3 Conclusions

In conclusion, a carbon-dot-based nano-strategy was provided to convert commercial FB-33 dye, in order to overcome the shortcomings of current FL dyes for fungal imaging. The resulting FB-CDs showed a significantly improved photobleaching resistance and an 8.6-fold increase in quantum yield over free FB-33. FB-CDs also displayed superstable fluorescence signals under various application conditions. In particular, FB-CDs exhibited a chitin-targeting capacity, and the benzenesulfonic acid functional groups on the surface of FB-CDs enabled them to rapidly and efficiently bind to chitin within the fungal cell wall, resulting in minimal background fluorescence, allowing high-performance wash-free fungal imaging within 30 s. This work supplies a new route for pathogenic microbial detection by a POCT way.

## 4 Methods

### 4.1 Materials and reagents

Fluorescent brightener 33 (FB-33), D-glucose and chitin were purchased from Shanghai Macklin Biochemical Co., Ltd. Ethanediamine (EDA) was acquired from Guangzhou Chemical Reagent Factory Co., Ltd. Citric acid (CA), sodium hydroxide and agar were purchased from Aladdin (Shanghai, China). Potassium chloride (KCl) was purchased from China National Pharmaceutical (Shanghai, China). X-10 PBS was purchased from biosharp (Hefei, China). All commercially available reagents were used directly without further purification. Ultrapure water (18.2 MΩ, AIC, USA) was used in all of the experiments.

### 4.2 Instruments

Fluorescence and ultraviolet visible (UV–vis) absorption spectra were recorded on a F-320 fluorescence spectrometer (GANGDONG, China) and a UV-6100 UV–visible spectrophotometer (MAPADA, China), respectively. Absolute quantum yield was measured by using an FLS-980 fluorescence spectrometer (Edinburgh Instruments, UK) with an integrated sphere. Transmission electron microscopic (TEM) images were taken on a JEM-2100F electron microscope (JEOL). Atomic force microscopy (AFM) images were taken on a Dimension Icon AFM (BURKER). Dynamic light scattering (DLS) and zeta potential were measured with a Malvern Zeta Sizer Nano (Malvern Instruments). X-ray diffraction (XRD) analysis was carried out on a D/Max 2500 V/PC X-ray diffractometer. X-ray photoelectron spectroscopy (XPS) was obtained with a Nexsa (Thermo Scientific). Fourier transform infrared (FT-IR) spectra were obtained in the 400–4000 cm^−1^ range using a Bruker ALPHA FT-IR spectrometer. Fluorescent imaging studies were performed with a HF2300L fluorescence microscope (HaoKang Biotechnology Co., Ltd, Guangzhou, China).

### 4.3 Preparation of FB-CDs and CA-CDs

FB-CDs were prepared based on the hydrothermal method. CA (5 g), ethylenediamine (4 mL), and FB-33 (0.5 g) were dissolved in 30 mL of deionized water. The solution was transferred to a poly (tetrafluoroethylene)-lined autoclave (50 mL) and then autoclaved at 180 °C for 8 h. It changed from colorless to brownish yellow, which indicated the formation of FB-CDs. The solution was cooled down to room temperature naturally and centrifuged at 10,000 rpm for 10 min to remove possible large particles. The supernatant was subjected to dialysis membrane (MWCO of 1000 Da) against deionized water for 24 h, then concentrated by a vacuum rotary evaporator to 10 mL, and finally, lyophilized to brown powders. As a control, CA-CDs were synthesized under similar conditions except adding FB-33 in reactants.

### 4.4 Fungus imaging with FB-CDs

*S. cerevisiae* was obtained from edible yeast powder. Yeast was cultured in yeast extract-peptone-dextrose (YPD) liquid medium at 28 °C and shaken at 160 rpm for 10 h. For fluorescent imaging, 5 μL of yeast liquid was mixed with 5 μL of 100 μg/mL FB-CDs solution for 30 s, then smeared on a microscope slide, and imaged under the HF2300L fluorescence microscope. *S. commune* was obtained from soil samples. The soil samples were diluted with ultra-pure water, added to YPD solid medium, and incubated at 28 °C for 1–3 days. Repeat the aforementioned experimental procedure while imaging under UV excitation. For simulated clinical sample preparation, a small amount of scurf was scraped from a volunteer’s foot, further mixed with *S. commune* for 5 min, and the sample was dropped on the glass slide for FL imaging.

## Supplementary Material

Supplementary Material Details
