# Supplementary material for: Fluorescein-derived carbon dots with chitin-targeting for ultrafast and superstable fluorescent imaging of fungi
Source: Nanophotonics. 2022 Sep 29;11(22):5121–31. doi: 10.1515/nanoph-2022-0468 (PMC11501690; doi:10.1515/nanoph-2022-0468)
Supplement: Supplementary file 1 — Supplementary Material Details [file j_nanoph-2022-0468_suppl.pdf]

# Electronic Supplementary Information

Ao Liu, Yiqiao Chen, Biwen Yang, Zhouyi Guo, Luoqi Mo, Haolin Chen, Chenglong Tao, Chengkang Su, Zhiming Liu*

# Fluorescein-derived carbon dots with chitin-targeting for ultrafast and superstable fluorescent imaging of fungi

***Corresponding author: Zhiming Liu,** MOE Key Laboratory of Laser Life Science & Guangdong Provincial Key Laboratory of Laser Life Science, College of Biophotonics, South China Normal University, Guangzhou 510631, China and Guangzhou Key Laboratory of Spectral Analysis and Functional Probes, College of Biophotonics, South China Normal University, Guangzhou 510631, China,
E-mail: liuzm021@126.com (Z. Liu)
**Ao Liu, Yiqiao Chen, Biwen Yang, Zhouyi Guo, Luoqi Mo,** MOE Key Laboratory of Laser Life Science & Guangdong Provincial Key Laboratory of Laser Life Science, College of Biophotonics, South China Normal University, Guangzhou 510631, China
**Haolin Chen,** Department of Hematology, The Seventh Affiliated Hospital, Sun Yat-sen University, Shenzhen 518107, China
**Chenglong Tao, Chengkang Su,** Guangzhou Haokang Biotechnology Co., Ltd., Guangzhou 510660, China

## Supplementary Figures

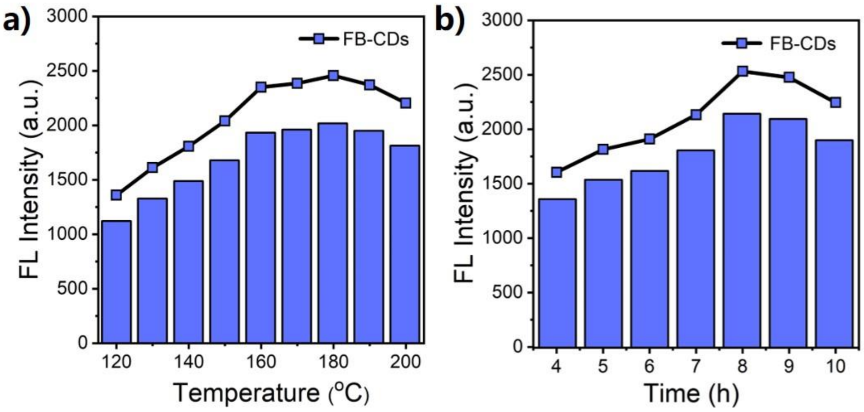


**Figure S1:** Fluorescent intensities of FB-CDs under different synthetic parameters: (a) hydrothermal temperature, (b) hydrothermal time.

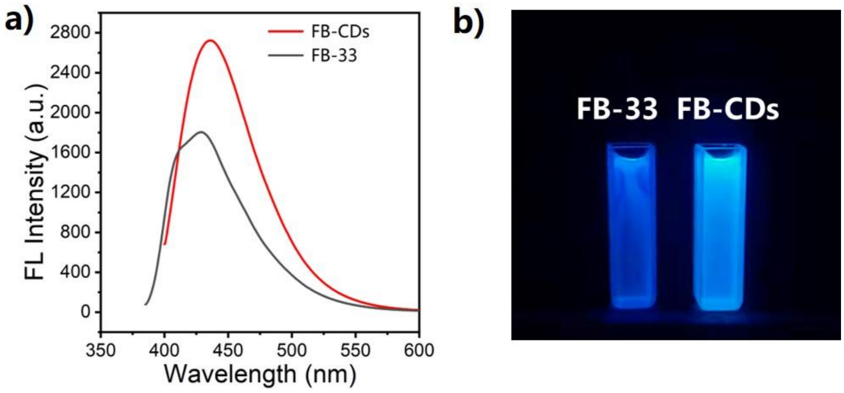


**Figure S2:** (a) Fluorescence spectra of FB-CDs and FB-33 at the same concentration (0.1 mg/mL). (b) Photographs of FB-33 and FB-CDs at the same concentration under UV light irradiation.

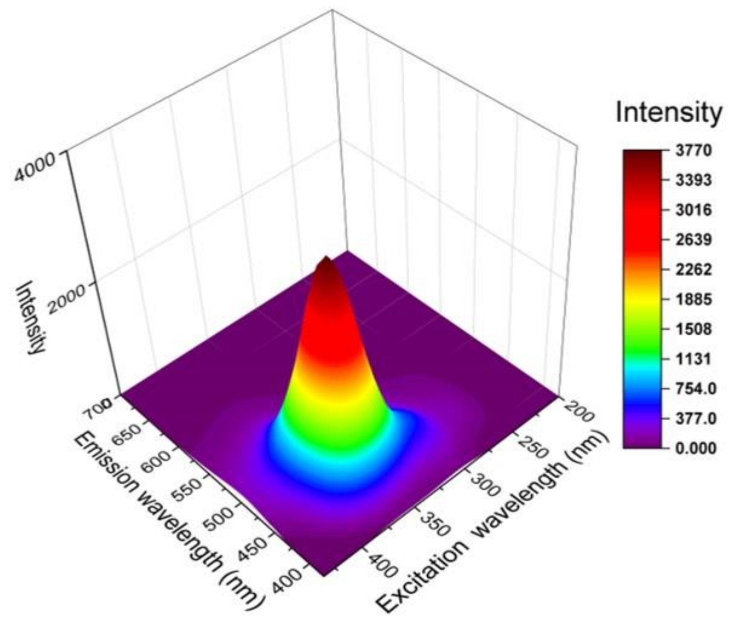


**Figure S3:** Three-dimensional fluorescence map of FB-CDs.

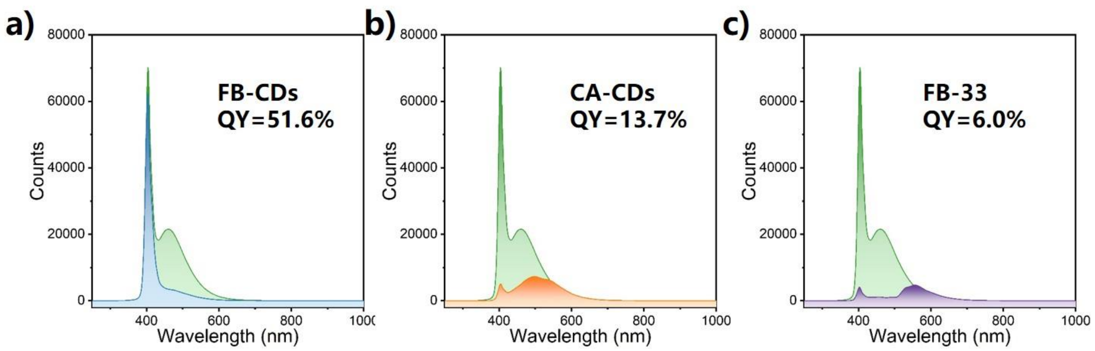


**Figure S4:** The absolute PLQY value of the FB-CDs, CA-CDs and FB-33.

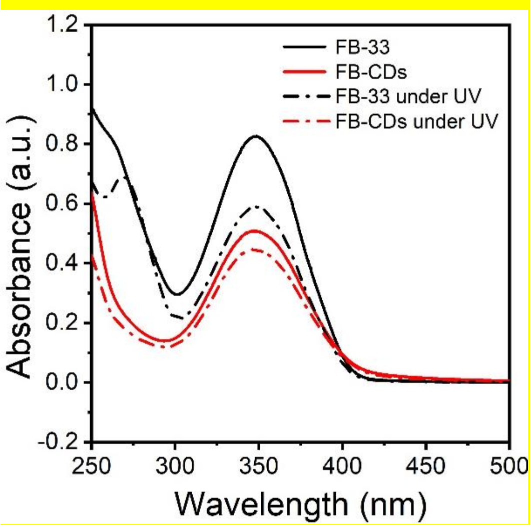


**Figure S5:** UV-vis absorption spectra of the FB-CDs and FB-33 before and after UV irradiation for 4 h.

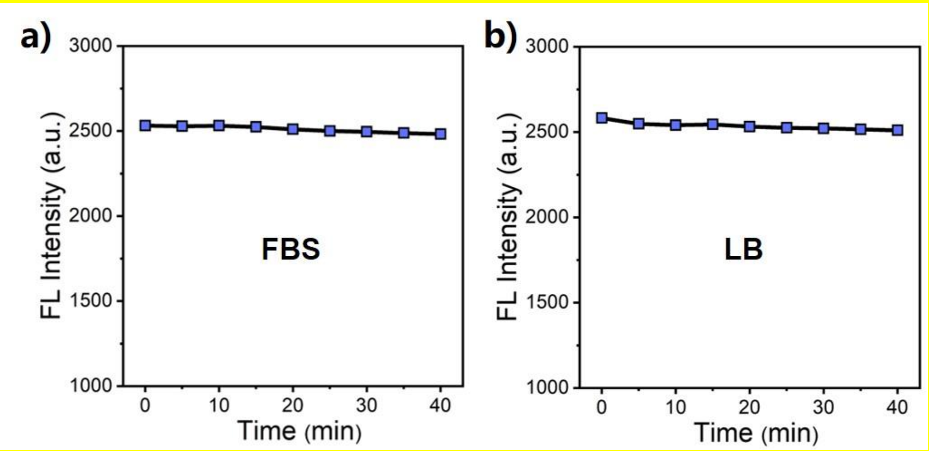


**Figure S6:** Fluorescence stability of FB-CDs dispersed in (a) FBS and (b) LB medium under continuous UV light irradiation for 40 min.

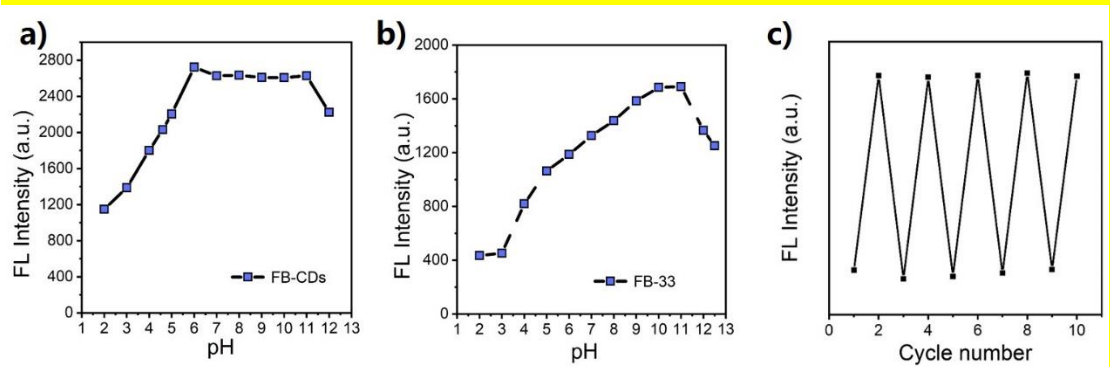


**Figure S7:** The changes of fluorescence intensities of (a) FB-CDs and (b) FB-33 solutions at different pH values (from 2 to 12). (c) Reversible fluorescence intensity of FB-CDs against pH from 3 to 11.

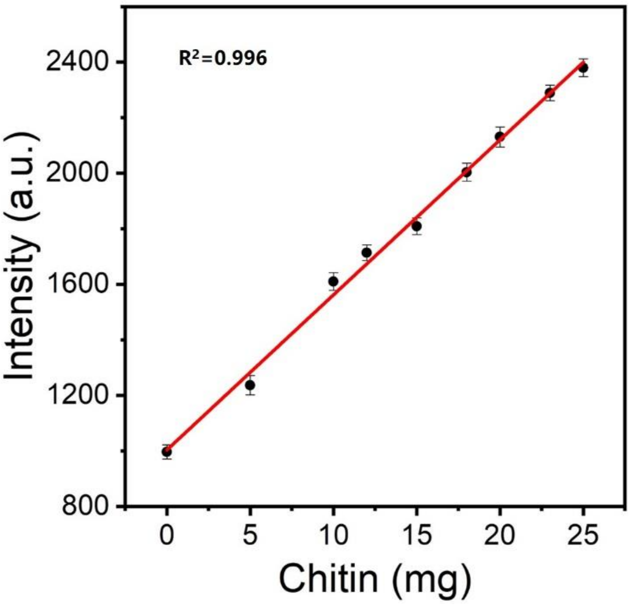


**Figure S8:** The fluorescence intensity of FB-33 (50 μg/mL) as a function of chitin mass.

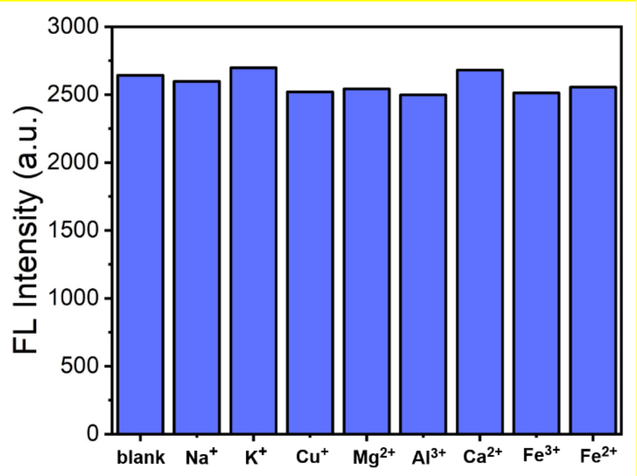


**Figure S9:** The influence of ion on the chitin-targeted capability of FB-CDs.

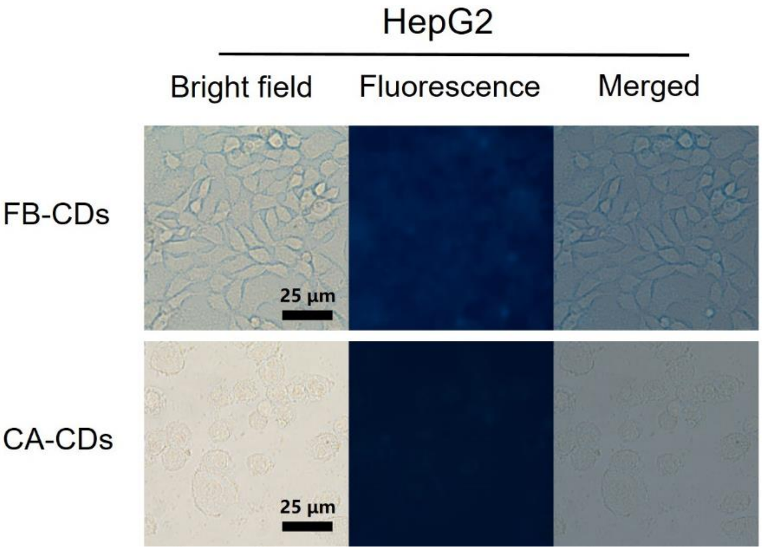


**Figure S10:** Fluorescence images of HepG2 cells treated with FB-CDs and CA-CDs at the same concentration (100 μg/mL) for 10 min.

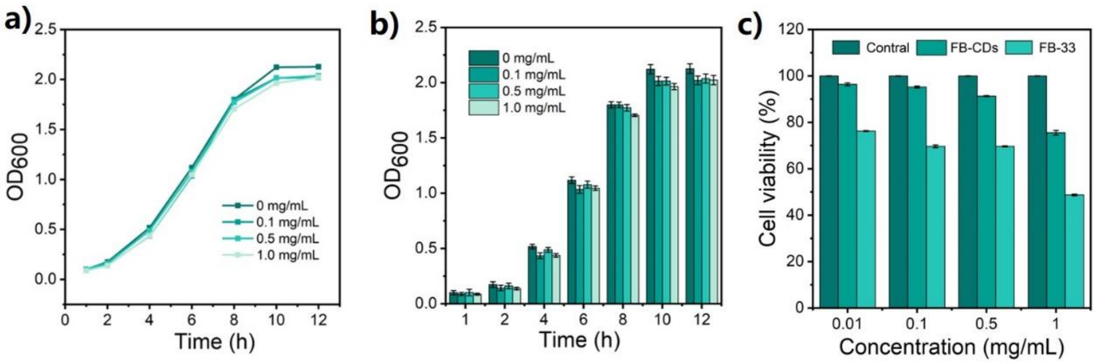


**Figure S11:** The biocompatibility of FB-CDs. (a) Growth curves of *S. cerevisiae* incubated with different concentrations of FB-CDs. The optical density value at 600 nm ($OD_{600}$) was recorded. (b) Statistical histograms corresponding to (a). (c) Relative viabilities of HepG2 cells after treatment with different concentrations of FB-CDs and FB-33 for 12 h.

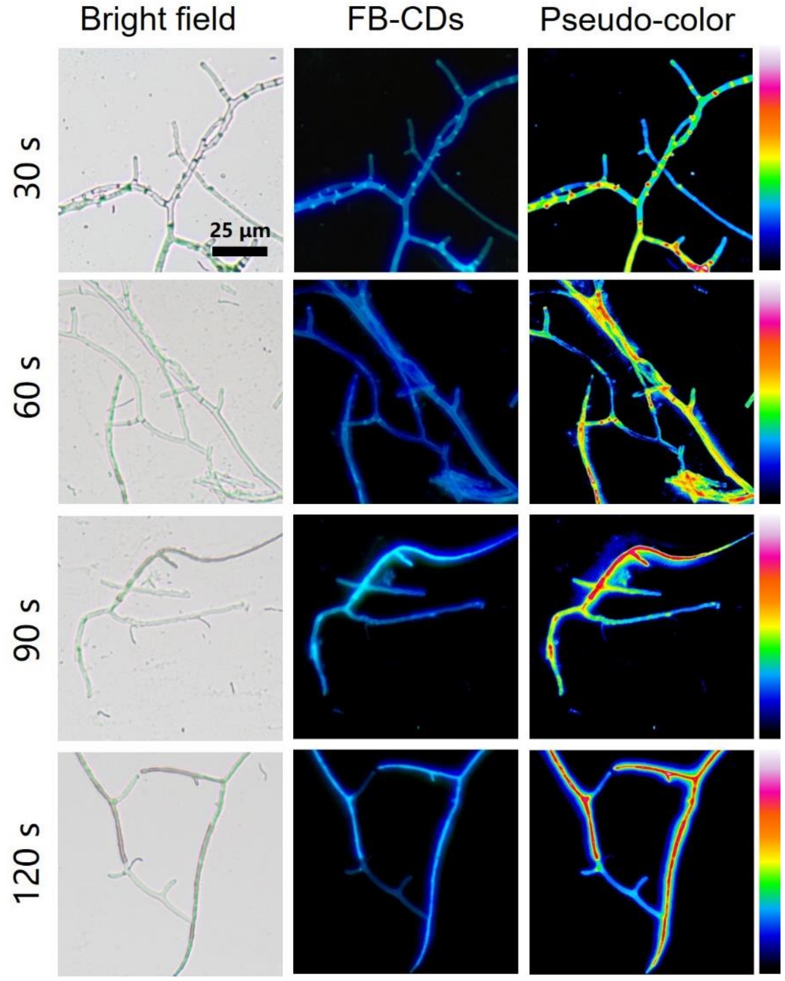


**Figure S12:** Fluorescence images of *schizophyllum commune* treated with FB-CDs (100 μg/mL) for different incubation times.

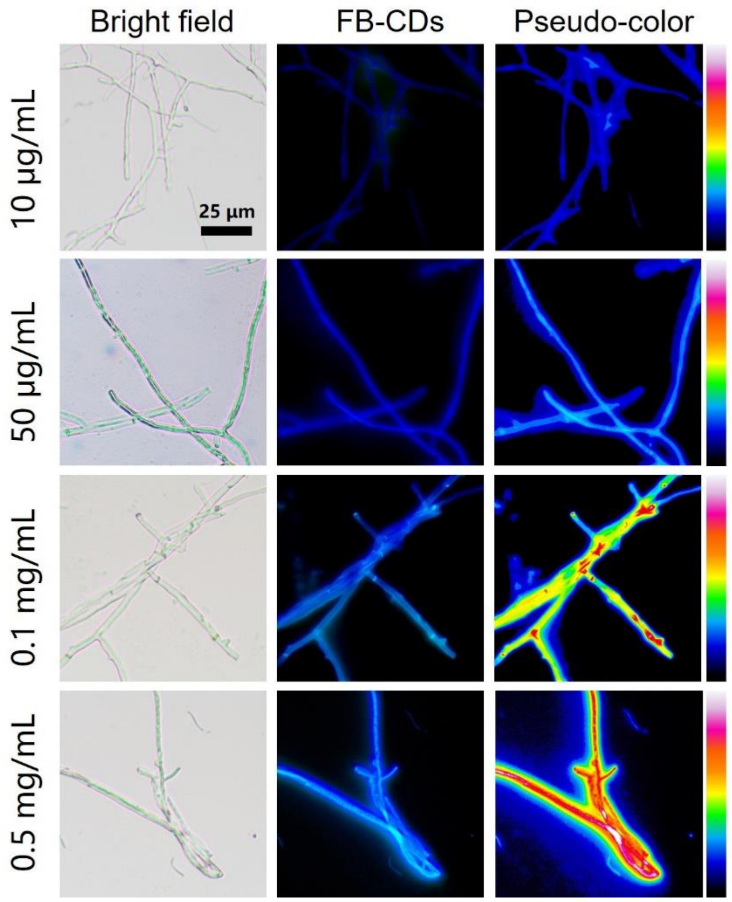


**Figure S13:** Fluorescence images of *schizophyllum commune* treated with different concentrations of FB-CDs for 30 s.

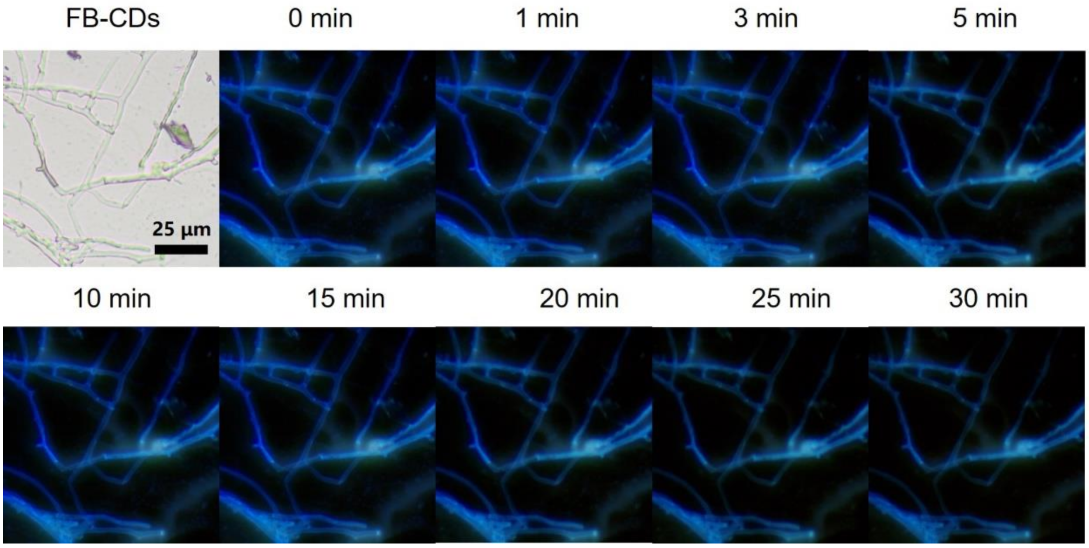


**Figure S14:** Long-term fluorescence images of *schizophyllum commune* incubated with FB-CDs for 30 s. The samples were exposed continuously for 30 min and the FL images were captured at different time points.

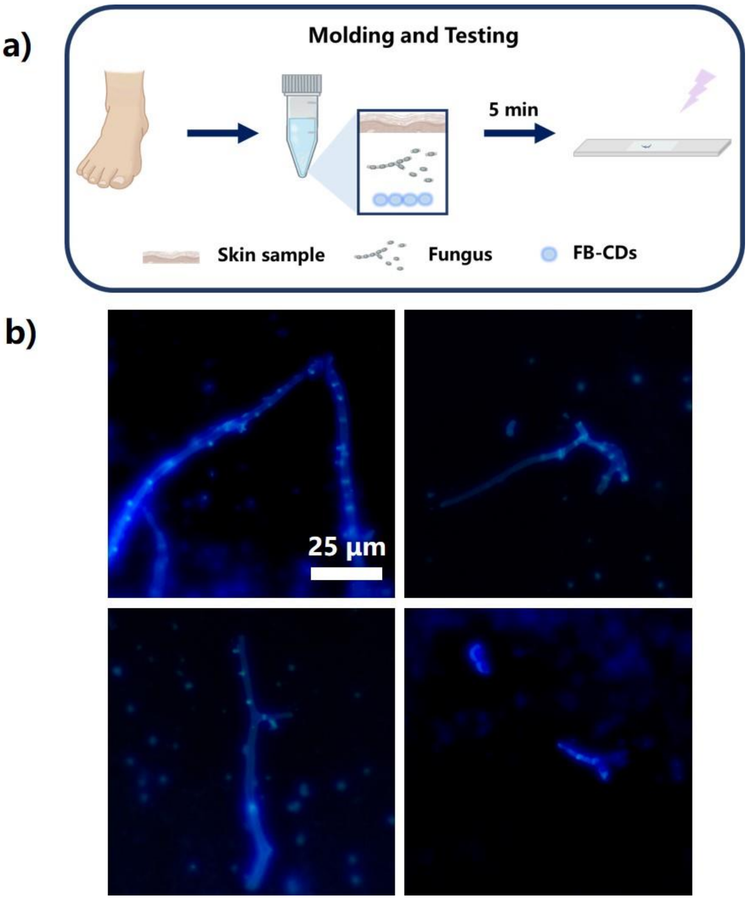


**Figure S15:** (a) Schematic illustration of the preparation of simulated clinical fungal sample. (b) Fluorescence images of simulated sample after treated with the FB-CDs.

# Supplementary Tables

**Table S1:** Comparison of FB-CDs with other fluorescent nanoagents.

| Materials | Staining time (min) | Photostability (min) | Emission (nm) | PLQY (%) | Ref. |
|---|---|---|---|---|---|
| s-N-CDs | unknown | 15 | 460 | 16 | [1] |
| BbimDCN-OCDs | 30 | unknown | 440 | 9.3 | [2] |
| CDs−$NH_2$ | 180 | 120 | 440 | 11.4 | [3] |
| CDs | 120 | 30 | 450 | 12.96 | [4] |
| CQDs-MNPs | 30 | 5 | 522 | 16.2 | [5] |
| CD-MB | 120 | 30 | 460 | 3.9 | [6] |
| OPDA nanodots | 360 | 120 | 450 | 6.4 | [7] |
| o-PDANPs | 120 | 120 | 455 | 1.4 | [8] |
| Au NCs | 240 | unknown | 580 | unknown | [9] |
| FB-CDs | 0.5 | 120 | 440 | 51.6 | This work |

**Table S2.** Comparison of FB-CDs with common fluorescent molecules for fungi imaging.

| Materials | Staining time (min) | Photostability (min) | Emission (nm) | Ref. |
|---|---|---|---|---|
| BFP protein | 24 h | Easy deactivation | 445 | [10] |
| CFP protein | 24 h | Easy deactivation | 480 | [11] |
| DAPI | 120 | Easy quenching | 461 | [12] |
| Calcofluor White | 120 | 1 | 472 | [13] |
| Dansyl dye | 60 | 2 | 520 | [14] |
| ER Tracker Green | 30 | 2 | 511 | [15] |
| FB-CDs | 0.5 | 120 | 440 | This work |

## References


[1] Yu Y.,Li C.,Chen C., et al. "Saccharomyces-derived carbon dots for biosensing pH and vitamin B 12". *Talanta*, vol. 195, pp. 117-126, 2019.

[2] Wang J. L.,Teng J. Y.,Jia T., et al. "Detection of yeast Saccharomyces cerevisiae with ionic liquid mediated carbon dots". *Talanta*, vol. 178, pp. 818-824, 2018.

[3] Zhang Y.,Zhao J.,Sun X., et al. "Fluorescent carbon dots for probing the effect of thiram on the membrane of fungal cell and its quantitative detection in aqueous solution". *Sens. Actuators B Chem.*, vol. 273, pp. 1833-1842, 2018.

[4] Yuan X.,Tu Y.,Chen W., et al. "Facile synthesis of carbon dots derived from ampicillin sodium for live/dead microbe differentiation, bioimaging and high selectivity detection of 2,4-dinitrophenol and Hg(II)". *Dyes Pigm.*, vol. 175, pp. 108187, 2020.

[5] Hu X.,Li Y.,Xu Y., et al. "Green one-step synthesis of carbon quantum dots from orange peel for fluorescent detection of Escherichia coli in milk". *Food Chem*, vol. 339, pp. 127775, 2021.

[6] Wu M.-S.,Zhou Z.-R.,Wang X.-Y., et al. "Dynamic Visualization of Endoplasmic Reticulum Stress in Living Cells via a Two-Stage Cascade Recognition Process". *Anal. Chem.*, vol. 94, pp. 2882-2890, 2022.

[7] Ma B.,Liu F.,Zhang S., et al. "Two-photon fluorescent polydopamine nanodots for CAR-T cell function verification and tumor cell/tissue detection". *J Mater Chem B*, vol. 6, pp. 6459-6467, 2018.

[8] Yin H.,Zhang K.,Wang L., et al. "Redox modulation of polydopamine surface chemistry: a facile strategy to enhance the intrinsic fluorescence of polydopamine nanoparticles for sensitive and selective detection of Fe3+". *Nanoscale*, vol. 10, pp. 18064-18073, 2018.

[9] Liang B.,Han L. "Displaying of acetylcholinesterase mutants on surface of yeast for ultra-trace fluorescence detection of organophosphate pesticides with gold nanoclusters". *Biosens. Bioelectron.*, vol. 148, pp. 111825, 2020.

[10] Loll-Krippleber R.,Feri A.,Nguyen M., et al. "A FACS-Optimized Screen Identifies Regulators of Genome Stability in Candida albicans". *Eukaryotic Cell*, vol. 14, pp. 311-322, 2015.

[11] Yanez-Carrillo P.,Orta-Zavalza E.,Gutierrez-Escobedo G., et al. "Expression vectors for C-terminal fusions with fluorescent proteins and epitope tags in Candida glabrata". *Fungal Genet. Biol.*, vol. 80, pp. 43-52, 2015.

[12] Brimacombe C. A.,Burke J. E.,Parsa J.-Y., et al. "A natural histone H2A variant lacking the Bub1 phosphorylation site and regulated depletion of centromeric histone CENP-A foster evolvability in Candida albicans". *PLOS Biol.*, vol. 17, 2019.

[13] Han Q.,Wang N.,Pan C., et al. "Elevation of cell wall chitin via Ca2+-calcineurin-mediated PKC signaling pathway maintains the viability of Candida albicans in the absence of beta-1,6-glucan synthesis". *Mol. Microbiol.*, vol. 112, pp. 960-972, 2019.

[14] Benhamou R. I.,Bibi M.,Steinbuch K. B., et al. "Real-Time Imaging of the Azole Class of Antifungal Drugs in Live Candida Cells". *ACS Chem. Biol.*, vol. 12, pp. 1769-1777, 2017.

[15] Jain P.,Sethi S. C.,Pratyusha V. A., et al. "Ras signaling activates glycosylphosphatidylinositol (GPI) anchor biosynthesis via the GPI–N-acetylglucosaminyltransferase (GPI–GnT) in Candida albicans". *J. Biol. Chem.*, vol. 293, pp. 12222-12238, 2018.
